# Feasibility and assessment of self-reported dietary recalls among newly diagnosed multiple sclerosis: a quasi-experimental pilot study

**DOI:** 10.3389/fnut.2024.1369700

**Published:** 2024-10-11

**Authors:** Solange M. Saxby, Mary A. Ehlinger, Lisa Brooks, Tyler J. Titcomb, Patrick Ten Eyck, Linda M. Rubenstein, Babita Bisht, Farnoosh Shemirani, Christine Gill, John Kamholtz, Linda G. Snetselaar, Terry L. Wahls

**Affiliations:** ^1^Department of Internal Medicine, University of Iowa, Iowa City, IA, United States; ^2^Department of Community and Family Medicine, Dartmouth Health, Lebanon, NH, United States; ^3^Institute for Clinical and Translational Science, University of Iowa, Iowa City, IA, United States; ^4^Department of Epidemiology, University of Iowa, Iowa City, IA, United States; ^5^Department of Neurology, University of Iowa Hospital and Clinics, Iowa City, IA, United States

**Keywords:** multiple sclerosis, modified Paleolithic diet, physical activity, mindfulness-based breathing, quasi-experimental

## Abstract

**Background:**

Individuals who are newly diagnosed with clinically isolated syndrome (CIS) or relapsing–remitting multiple sclerosis (RRMS) may choose not to undergo disease-modifying therapies (DMTs) due to concerns about expenses or potential adverse effects. Thus, many individuals will opt for alternative therapies, such as dietary modifications. Among these dietary approaches, the modified Paleolithic elimination diet has shown promise for improving MS-related symptoms; however, restriction of certain food groups can lead to inadequate intake of nutrients.

**Methods:**

Three-day self-reported 24-h dietary recalls using the Automated Self-Administered 24-h (ASA24) Dietary Assessment Tool were assessed during a 12-month quasi-experimental (i.e., non-randomized) trial among individuals who either voluntarily declined DMTs and received health behavior (HB) intervention, (*n* = 29) or included DMTs and opted for the standard of care (SOC; *n* = 15). Participants in the HB group received a multimodal intervention that included dietary modifications, a walking program, and breathing exercises. Usual intake of each micronutrient was estimated and then evaluated with the estimated average requirement (EAR)-cut point method.

**Results:**

At 12 months, >80% of both HB and SOC groups completed 3 days of the self-reported 24-h recalls, indicating the potential feasibility of ASA24. From baseline to 12 months, the HB group had a decreased mean ratio in total grains (0.64; 95% CI 0.43–0.93; *p* = 0.02) and added sugars (0.52; 95% CI 0.35–0.75; *p* ≤ 0.001), and an increased mean ratio intake of cured meats (1.74; 95% CI 1.05–2.90; *p* = 0.04); whereas, the SOC group had a decreased mean ratio intake for beef, veal, pork, lamb, and game meat (0.60; 95% CI 0.40–0.90; *p* = 0.01). At baseline, both groups had high proportions with inadequate intake of vitamin E and calcium. The SOC group also had a high proportion with inadequate intake of vitamin D. By 12-months, the HB group exhibited severe proportions of nutrient inadequacies (>20% of the group) for vitamin D (43.5%), vitamin E (29.1%), calcium (69.9%), and copper (27.8%). The SOC group, following their own diet, had inadequacies for all the same micronutrients, except for copper, as the HB group. The SOC group also had additional inadequacies: vitamin A (21.3%), thiamin (26.3%), riboflavin (24.2%), folate (24.8%), vitamin B12 (27.8%), and zinc (28.2%).

**Conclusion:**

Compared to the usual diet, adhering to the modified Paleolithic elimination diet, as a component of a 12-month multimodal intervention, may lead to reduced consumption of specific food groups, such as added sugars, as well as decreased risk of severe proportions of inadequacy for certain nutrients. The utilization of the ASA24 for acquiring dietary recalls from participants with MS may be feasible for future studies.

**Clinical trial registration:**

clinicaltrials.gov identifier NCT04009005.

## 1 Introduction

Multiple sclerosis (MS) is a neurodegenerative disease of the central nervous system characterized by demyelination and inflammation (1). Newly diagnosed individuals often develop a single demyelinating event of the central nervous system, termed clinically isolated syndrome (CIS); with a subsequent demyelinating event, called a relapse, the individual can be described as having relapsing–remitting MS (RRMS) (2). Following a new diagnosis, standard of care (SOC) practices suggest starting a pharmacological disease-modifying therapy (DMT) for individuals with CIS, with unfavorable prognostic factors, or RRMS, with at least one relapse in the previous 2 years (3). While DMTs are efficacious and FDA-approved for reducing relapse risk and slowing disease progression (4); some individuals diagnosed with MS may voluntarily decline DMTs due to the high costs (5), concerns about adverse effects (6), or a lack of health insurance coverage (7). As such, there is a growing interest in more holistic approaches to treatments, such as dietary modifications, among individuals with MS (8). Therefore, there is a need to understand the effect of alternative or adjunctive programs among people with MS who are voluntarily DMT-naïve on their clinical progression.

People with MS have expressed great interest in diet (8) and a desire for evidence-based dietary guidelines and support for better managing symptoms during the MS disease course (9–12). While evidence for specific therapeutic diets has been inconsistent (13), recent meta-analyses (14, 15) of randomized dietary intervention trials in MS found that several dietary interventions, including the modified Paleolithic diet, may lead to improved quality of life (QoL) and reduced fatigue. The modified Paleolithic elimination diet, based on Paleolithic principles, eliminates specific dietary antigens (gluten, casein, and lectins) and enhances micronutrient density (16). Previous modified Paleolithic elimination diet intervention studies have demonstrated favorable improvements among individuals with MS for MS-related symptoms, including fatigue and QoL (17, 18). Given the restrictive intake of food groups on the modified Paleolithic elimination diet, people with MS have potential risks related to nutrient deficiencies. Inadequate intake of certain nutrients, such as folate, magnesium, and vitamin D, are associated with more severe MS symptoms (19, 20). Thus, ensuring individuals following the modified Paleolithic elimination diet are meeting their nutrient requirements is of great importance.

Previous studies have explored the nutrient content of the modified Paleolithic elimination diet (21). Examination of experimental menus for the modified Paleolithic diet suggested its sufficiency in meeting all micronutrient requirements, except for vitamin D, calcium, potassium, and choline across various life stage groups, as well as iron among women of reproductive age (16). In addition, in a randomized control trial of dietary intervention, a three-day weighed food record analysis revealed that adhering to the modified Paleolithic elimination diet had significant reductions in the proportion with inadequate intake of food for calcium, thiamin, and vitamin B_12_ (22). Similarly, in a pilot study, individuals with MS who reported following the Paleolithic diet were found to be below the estimated adequacy ratio (EAR) for vitamins E and D (23). Therefore, the aim of this study was to evaluate food groups and nutrient intakes among newly diagnosed individuals with CIS or RRMS, who are following the modified Paleolithic elimination diet as part of a remote multimodal intervention, compared to individuals following their usual diet and standard of care, which includes DMTs. In addition, although various methods have been used to assess dietary intake among people with MS, there is a need to assess the feasibility of an online self-reported dietary recall methodology among newly diagnosed people with CIS and RRMS.

## 2 Methods

### 2.1 Study design

The study design and primary outcome of QoL results have been described elsewhere (24). In brief, this was a 12-month, quasi-experimental trial conducted at the Prevention Intervention Center at the University of Iowa Hospitals and Clinics. Given the ethical concerns of withholding FDA-approved DMTs from individuals with MS, a quasi-experimental study design was selected to avoid randomization of participants into a non-DMT treatment group (25). Individuals newly diagnosed with CIS and RRMS who followed the standard of care (SOC group), which included DMTs, were compared to individuals who voluntarily declined DMTs and were eligible to receive a multimodal health behavior (HB group) modification intervention that was comprised of diet, a walking program, and breathing exercise. Prior to enrollment, informed consent was obtained from all participants. The University of Iowa Institutional Review Board approved the study protocol (IRB #201908778). The study is registered at clinicaltrials.gov, with the identifier: NCT04009005.

### 2.2 Participants

Participants were recruited from the continental United States through social media posts, email blasts, and flyers sent to local neurology clinics, and were eligible for enrollment if they had: (1) a diagnosis of RRMS or CIS according to the 2017 McDonald criteria (2), (2) confirmed by the treating neurologist no more than 12 months before the first study visit was completed; (3) between 18 and 55 years of age at the time of consent; (4) consent to share the clinical notes from the primary care and neurology providers during the study period; (5) residence within the continental United States; (6) approval of enrollment by the treating neurologist. Major exclusion criteria for all participants included: (1) moderate or severe mental impairment as measured by the Short Portable Mental Health Questionnaire (26); (2) use of insulin or Coumadin medication; (3) history of oxalate kidney stones, schizophrenia, or active diagnosis of an eating disorder. The complete list of inclusion and exclusion criteria can be found elsewhere (24).

### 2.3 Study procedures

Upon enrollment, participants in the HB group (*n* = 29) received the multimodal intervention, which consisted of the following components: a modified Paleolithic elimination diet, 4–7–8 breathing exercises, and a moderate-intensity walking program. At baseline, the participants were provided with an educational module that encompassed an in-depth exploration of the interplay between diet, aerobic exercise, and stress reduction mechanisms, elucidating their potential impacts on both patient-reported symptoms and concurrent comorbid disease processes. Subsequently, the participants were contacted by the study-registered dietitian nutritionist (RDN; LB), who is trained in motivational interviewing and self-determination theory (27–29), to provide an orientation session via Zoom or video call on the study’s intervention diet, walking program, and stress reduction. The RDN answered questions and consulted with the study PI (TW), physical therapist (PT; BB), and other study team members regarding the walking or stress reduction components and motivation for sustaining the behavior changes required by the study. Upon completion of the first month, the RDN scheduled monthly video group support calls or individual coaching calls, based on the participant’s preference. Participants received emails and text support from the study RDN, as needed, as well as regular group emails.

In contrast, the SOC group (*n* = 15) did not receive any guidance or instruction regarding dietary or behavioral interventions. Nonetheless, to sustain participants’ involvement with the research, the SOC group received monthly emails containing updates on the most recent MS research unrelated to diet, physical activity, or mindfulness practices. The study team did not impose any limitations on the diets or behavior-related endeavors of SOC participants.

### 2.4 Intervention diet and supplements

The study RDN provided guidance to the HB group on following the modified Paleolithic elimination diet. The study diet emphasized consuming 6–9 servings of combined fruits and vegetables daily, along with 9–12 ounces of meat for petite women and 12–21 ounces of meat for men and tall women, adjusting according to the individual’s gender and size. Notably, the study diet excluded all gluten-containing grains, legumes, eggs, and dairy, with the exception of clarified butter or *ghee*. Additionally, nightshade vegetables, such as tomatoes, white potatoes, eggplants, peppers, as well as seeds and spices, were eliminated from months 3 to 6 of the diet. If desired by the participants, the reintroduction of nightshades, as well as seeds and spices, occurred during months 7–9, with the process closely supervised by the research team to assess tolerance and offer recommendations if adjustments were needed. Following the identification of problematic foods during months 7–9, participants proceeded to follow a personalized modified Paleolithic elimination diet during months 10–12.

In addition to dietary modification, participants were also recommended to take the following supplements: fish oil (2 g), vitamin B12/methyl folate/pyridoxyl-5-phosphate (1,000 mcg/400 mcg/1.5 mg per day, respectively), vitamin D3 (2,500 IUs per day), N-acetyl-l-cysteine (500 mg per day), phosphatidylcholine (420 mg).

### 2.5 Three-day automated self-administered dietary recalls

Developed by the National Cancer Institute (NCI), the Automated Self-Administered 24-Hour Dietary Assessment Tool (ASA24; version 2020) (30), was utilized to assess the participant’s dietary intake through a secure online website. The ASA24 is a self-administered multi-pass 24-h recall tool for recalling food intake from the prior 24-h period, where participants are led through a series of steps to document all the food and drinks consumed in the past 24 h. Detailed information about the initial validation study for ASA24 has been previously published (31, 32).

The Study Coordinator (MAE) sent an email at the scheduled times to each participant (baseline, months 3, 6, 9, and 12), which included the website for the ASA24 Recalls^1^ and the participant’s login information. These recalls were to be completed on three non-consecutive days, with two during weekdays (Monday to Thursday), and one during the weekend (Friday to Sunday). In addition, the Study Coordinator monitored the completion of the recalls by logging into the ASA24 website and seeing if participants had been able to complete the recalls. On occasion, when a participant was having trouble with the program, the study RDN was asked to log into a specific participant’s account to troubleshoot the issue or complete the recalls over the phone. The study RDN also contacted NCI support for assistance with issues. In addition, due to the lack of certain foods or ingredients consumed in the study diet, participants were provided with suggested food substitutions. For instance, any type of leafy green vegetable eaten would be entered in the ASA24 as the variable “greens (other kind)”. A list of food alternatives provided to participants can be found in Supplementary Table 1.

Data from the recalls was not used for diet intervention coaching and was not analyzed until the conclusion of the study. Instead, the self-monitoring smartphone application, in which answers are integrated into the respective Research Electronic Data Capture (REDCap) (33, 34), a secure web-based software platform designed to support data capture for research studies, was used to assist the study RDN in coaching individuals and groups regarding their ongoing diet adherence. During the monthly remote group meetings via Zoom, HB group adherence averages were presented, providing group-level feedback on food intake. Subsequently, each participant received their personal averages via a private email message. Specific counseling sessions related to individual participant averages were not conducted.

### 2.6 Statistical analysis

Baseline characteristics of treatment groups were summarized using counts and percentages or means and standard deviations. Between-treatment comparisons were made using Fisher’s exact test for categorical variables and using a two-sample t-test for continuous variables. Feasibility of the ASA24 assessment (i.e., the number of recalls completed by each participant) was reported as study-time stratified counts and percentages. The number of recalls completed for the HB and SOC groups was assessed for differences using Fisher’s exact test.

ASA24 dietary food and supplement recall data was checked for accuracy and possible entry errors. Based on previous research (35), any days in which implausible energy intake below 500 kcal/day or above 4,000 kcal/day were considered erroneous and were excluded. The mean intake of each nutrient from the ASA24 dietary recalls at each study time point (i.e., months 0, 3, 6, 9, and 12) was calculated for each participant and adjusted for age, gender, and BMI, as well as weekday vs. weekend recall values using the NCI method to estimate the usual intake (36). While 3-day 24-h recalls were requested from participants at each time point, the NCI Method handles missing 24-h dietary recalls by using analytical techniques to estimate the distribution of usual intake even when only 1 or 2 days of 24-h recalls are available. Within- and between-treatment changes in outcomes over time were tested using the generalized linear mixed modeling (GLMM) (37) framework. All models include fixed effects for the treatment group, time, and their interaction. The models also specify a random effect for participants to account for repeated measures. Point estimates, 95% confidence intervals, and *p*-values for within- and between-treatment mean differences/ratios over time were generated for each outcome. Graphical observation was used to evaluate the normality of data. If data had moderately to severely right-skewed distributions, a negative binomial distribution was used to assess mean change over time.

The usual intake of each nutrient was then compared to the estimated average requirement (EAR) for each life stage group using the EAR-cut point method (38) and combined by weighted means to assess the proportion of each group with inadequate micronutrient intake. Bioconversion of provitamin A carotenoids was accounted for by using retinol activity equivalents (RAEs) for vitamin A. The combination of ergocalciferol (vitamin D_2_) and cholecalciferol (vitamin D_3_) was used to assess vitamin D. The bioconversion of tocopherols was accounted for by using alpha-tocopherol equivalents for vitamin E. To account for the differences in the absorption of food folate and of synthetic folic acid obtained from dietary supplements or food fortified with folic acid, Dietary Folate Equivalents (DFE) were used to assess folate (39). Niacin equivalents (NE) were used to assess niacin, which accounts for the contribution of dietary intake of all the forms of niacin that are available to the body, such as tryptophan (40). Given that iron recommendations are not normally distributed among women of reproductive age and are different for postmenopausal women, the proportion of iron inadequate intake was estimated using the probability approach recommended by the National Academy of Medicine (formerly the Institute of Medicine) (41). As previously described (42), the threshold for severe inadequate intake of micronutrients was defined as ≥20% of the group. Usual intake was also compared to Tolerable Upper Intake Levels (ULs) to determine the proportion of excessive intakes.

All analyses were performed with two-sided tests (*α* = 0.05) using SAS software (version 9.4, SAS Institute, Inc.) and Microsoft Excel (Version 16.86).

## 3 Results

At baseline, 29 individuals enrolled in the HB group (*n* = 29) and 15 in the SOC (*n* = 15) groups, for a total of 44 participants in the trial. At baseline, none of the characteristics assessed differed significantly between the groups (Table 1).

**Table 1:** Baseline characteristics of study participants in the health behaviors (HB) and standard of care (SOC) groups.^1^

Characteristic	Health behaviors	Standard of care	*p*-value^2^
*n*	29	15	
Age (years)	38.0 ± 1.1	41.1 ± 2.3	0.19
Female (% males)	26 (89.7%)	15 (100%)	0.54
MS duration (years)	0.29 ± 0.04	0.34 ± 0.08	0.57
BMI	25.80 ± 1.0	24.3 ± 1.0	0.32
Race			
White	23 (79.3%)	13 (86.7%)	>0.99
Black	1 (3.5%)	0	
Latin or Hispanic	1 (3.5%)	0	
Two or more races	2 (6.9%)	1 (6.7%)	
Unknown or Not Reported	2 (6.9%)	1 (6.7%)	

Adapted from (24).

BMI, body mass index; MS, multiple sclerosis.

1 Data are shown as mean ± SE or *n* (%).

2 Significance determined using Fisher’s exact test for categorical variables or two-sample t-test for continuous variables.

Baseline food group equivalent mean intakes were compared to within-group average consumption during the study time point intervals (Table 2). At the 12-month primary endpoint, the HB group exhibited a decreased intake of total grains (Mean Ratio 0.64; 95% CI 0.43–0.93; *p* = 0.02) and added sugars (Mean Ratio 0.52; 95% CI 0.35–0.75; *p* ≤ 0.001) and an increased intake of cured meats (Mean Ratio 1.74; 95% CI 1.05–2.90; *p* = 0.04). Notably, added sugars were the only food group equivalent in which the mean intake had significantly decreased from baseline in the HB group throughout all the time point intervals (i.e., months 3, 6, 9, and 12, *p* < 0.05 for all) during the study (Supplementary Table 2). Conversely, the SOC group demonstrated a decrease in food group equivalents from baseline to 12 months only for beef, veal, pork, lamb, and game meat (Mean Ratio 0.60; 95% CI 0.40–0.90; *p* = 0.01). The only significant between-group assessments of intake change from baseline to 12-months were observed in other starchy vegetables (excludes white potatoes) (*p* = 0.04), total whole grains (*p* = 0.05), and beef, veal, pork, lamb, and game meat (*p* = 0.03).

**Table 2:** Food group equivalent outcomes at baseline and 12-month means and absolute/proportional mean change for the health behaviors (HB) intervention and standard of care.

	Health behavior			Standard of care			HB *vs* SOC
	Baseline Mean ± SE^1^	Month 12 Mean ± SE^1^	Mean Diff/Ratio (95% CI)^2^	Baseline Mean ± SE^1^	Month 12 Mean ± SE^1^	Mean Diff/Ratio (95% CI)^2^	*p*-value^3^
Fruits categories (cup eq.)							
Total fruits	1.55 ± 0.15	1.52 ± 0.15	0.98 (0.69–1.39)	1.85 ± 0.23	2.12 ± 0.24	1.15 (1.15–1.52)	0.86
Whole or cut citrus, melons, berries (excludes juices)	0.64 ± 0.09	0.59 ± 0.08	0.91 (0.59–1.40)	0.48 ± 0.10	0.58 ± 0.14	1.22 (0.76–1.95)	0.37
Whole or cut other fruits (excludes juices)	0.73 ± 0.09	0.75 ± 0.12	1.02 (0.62–1.70)	0.94 ± 0.16	0.93 ± 0.15	0.99 (0.60–1.63)	0.93
Fruit juices	0.18 ± 0.03	0.19 ± 0.04	1.07 (0.57–2.00)	0.44 ± 0.13	0.61 ± 0.22	1.40 (0.87–2.26)	0.50
Vegetables categories (cup eq.)							
Total vegetable (excludes legumes)	4.71 ± 0.37	5.14 ± 0.38	1.09 (0.90–1.33)	3.82 ± 0.40	3.67 ± 0.36	0.96 (0.71–1.31)	0.49
Dark green vegetables	1.74 ± 0.24	2.24 ± 0.24	1.29 (0.98–1.69)	1.15 ± 0.18	1.18 ± 0.15	1.03 (0.73–1.44)	0.31
Total red and orange vegetables	0.71 ± 0.08	0.66 ± 0.08	0.94 (0.72–1.23)	0.69 ± 0.13	0.59 ± 0.09	0.86 (0.54–1.35)	0.72
Tomatoes and tomato products	0.21 ± 0.03	0.19 ± 0.04	0.93 (0.53–1.63)	0.43 ± 0.09	0.22 ± 0.05	0.51 (0.25–1.03)	0.19
Other red and orange vegetables (excludes tomatoes)	0.50 ± 0.07	0.47 ± 0.07	0.94 (0.62–1.43)	0.26 ± 0.07	0.37 ± 0.08	1.43 (0.92–2.21)	0.18
Total starchy vegetables	0.22 ± 0.05	0.21 ± 0.05	0.96 (0.58–1.60)	0.33 ± 0.08	0.35 ± 0.10	1.04 (0.44–2.45)	0.89
White potatoes	0.18 ± 0.05	0.12 ± 0.03	0.69 (0.38–1.26)	0.26 ± 0.08	0.31 ± 0.10	1.18 (0.45–3.05)	0.35
Other starchy vegetables (excludes white potatoes)	0.04 ± 0.02	0.09 ± 0.04	2.07 (0.80–5.33)	0.07 ± 0.02	0.04 ± 0.02	0.55 (0.25–1.23)	**0.04**
Other vegetables not listed above	2.04 ± 0.18	2.02 ± 0.21	0.99 (0.73–1.34)	1.65 ± 0.28	1.56 ± 0.23	0.94 (0.58–1.55)	0.87
Legumes (beans and peas) computed as vegetables	0.06 ± 0.02	0.02 ± 0.01	0.31 (0.08–1.15)	0.10 ± 0.03	0.13 ± 0.06	1.33 (0.40–4.43)	0.11
Grain category (oz. eq.)							
Total grains	1.98 ± 0.27	1.26 ± 0.21	0.64 (0.43–0.93)*	2.26 ± 0.36	2.78 ± 0.54	1.23 (0.64–2.36)	0.08
Total whole grains	0.12 ± 0.04	0.24 ± 0.07	1.95 (0.89–4.24)	0.46 ± 0.13	0.33 ± 0.11	0.71 (0.38–1.33)	**0.05**
Total refined grains	1.86 ± 0.26	1.02 ± 0.19	0.55 (0.34–0.89)	1.79 ± 0.33	2.45 ± 0.53	1.37 (0.61–3.06)	0.06
Protein category (oz. eq.)							
Total protein foods (excludes legumes)	9.61 ± 0.60	10.2 ± 0.70	1.05 (0.85–1.28)	8.06 ± 1.09	5.96 ± 0.59	0.76 (0.51–1.14)	0.17
Beef, veal, pork, lamb, game meat	2.23 ± 0.39	2.50 ± 0.46	1.12 (0.74–1.69)	1.51 ± 0.40	0.91 ± 0.32	0.60 (0.40–0.90)*	**0.03**
Cured meats	0.53 ± 0.12	0.92 ± 0.29	1.74 (1.05–2.90)*	0.25 ± 0.07	0.73 ± 0.35	2.95 (0.73–11.91)	0.49
Organ meat^5^	0.12 ± 0.06	0.22 ± 0.10	0.09 (−0.07 to 0.26)	0.00 ± 0.00	0.00 ± 0.00	0.00 (0.00–0.00)	0.27
Poultry	2.52 ± 0.44	2.89 ± 0.45	1.15 (0.73–1.81)	1.48 ± 0.48	1.75 ± 0.44	1.19 (0.57–2.46)	0.94
Eggs and egg substitutes	0.21 ± 0.06	0.38 ± 0.12	1.80 (0.77–4.20)	0.25 ± 0.09	0.19 ± 0.10	0.77 (0.23–2.65)	0.27
Soy products excluding soy milk^4^	0.02 ± 0.02	0.06 ± 0.03	0.04 (−0.6 to 0.13)	0.21 ± 0.10	0.32 ± 0.18	0.11 (−0.17–0.39)	0.63
Peanuts, tree nuts, seeds (excludes coconut)	0.24 ± 0.08	0.08 ± 0.05	1.00 (0.57–1.73)	0.40 ± 0.13	0.54 ± 0.23	0.60 (0.34–1.05)	0.21
Dairy category (cup eq.)							
Total dairy	0.54 ± 0.08	0.28 ± 0.06	0.52 (0.29–0.94)	0.78 ± 0.12	0.58 ± 0.12	0.75 (0.42–1.35)	0.38
Milk (includes calcium-fortified soy milk)	0.25 ± 0.04	0.14 ± 0.03	0.54 (0.24–1.22)	0.45 ± 0.09	0.28 ± 0.07	0.61 (0.33–1.16)	0.80
Yogurt	0.07 ± 0.03	0.02 ± 0.01	0.30 (0.06–1.59)	0.04 ± 0.02	0.02 ± 0.02	0.49 (0.14–1.76)	0.65
Cheese	0.18 ± 0.05	0.10 ± 0.04	0.54 (0.16–1.78)	0.21 ± 0.08	0.15 ± 0.05	0.75 (0.23–2.42)	0.70
Other							
Added sugars (tsp. eq.)	7.46 ± 0.76	3.84 ± 0.56	0.52 (0.35–0.75)***	8.78 ± 1.57	7.04 ± 0.89	0.80 (0.50–1.35)	0.16
Alcoholic beverages (no. of drinks)	0.26 ± 0.08	0.12 ± 0.04	0.45 (0.19–1.08)	0.70 ± 0.28	0.48 ± 0.18	0.55 (0.30–1.00)	0.71

1 Data are shown as mean ± standard error (SE).

2 Absolute/proportional mean change from baseline to 12-months.

3 Significance was determined using generalized linear models with an identity or negative binomial link function.

4 Data normally distributed.

Within-group statistical significance compared to baseline values indicated by ^*^ for (*p* ≤ 0.05), ^**^ for (*p* ≤ 0.01), and ^***^ for (*p* ≤ 0.001).

Bold values indicate within-group statistical significance.

Baseline nutrient intake from food and supplements was evaluated, followed by subsequent within-group comparisons to the study time point intervals (Table 3). By the 12-month primary endpoint, the HB group exhibited a decreased intake of carbohydrates (0.83; 95% CI 0.70–0.99, *p* = 0.04) and vitamin D (0.54; 95% CI 0.31–0.0.3; *p* = 0.03). In comparison, the SOC group observed decreases in energy (0.84; 95% CI 0.71–0.99; *p* = 0.04), total fat (0.73; 95% CI 0.62–0.86; *p* = 0.0001), vitamin E (0.78, 95% CI 0.65–0.94; *p* = 0.01). Additional mean nutrient intake from food and supplements for months 3, 6, and 9 can be found in the supplemental materials (Supplementary Table 3). The only significant between-group assessments of intake change from baseline to 12-month were observed for total fat (*p* = 0.01), vitamin E (*p* = 0.04), and choline (*p* = 0.04).

**Table 3:** Food and supplement intake from baseline and 12-month means and proportion of mean change for the health behaviors (HB) intervention and standard of care.

	Health behavior			Standard of care			HB *vs* SOC
	Baseline Mean ± SE^1^	Month 12 Mean ± SE^1^	Mean Ratio (95% CI) ^2^	Baseline Mean ± SE^1^	Month 12 Mean ± SE^1^	Mean Ratio (95% CI)^2^	*p*-value^3^
Macronutrients							
Energy (kcal/day)	1,832.09 ± 63.20	1,679.07 ± 69.75	0.91 (0.81–1.04)	1,783.11 ± 104.04	1,509.09 ± 81.98	0.84 (0.71–0.99)*	0.42
Protein (g/day)	90.03 ± 4.06	89.58 ± 4.74	1.00 (0.87–1.13)	76.65 ± 7.41	65.45 ± 4.59	0.85 (0.65–1.13)	0.33
Carbohydrate (g/day)	168.58 ± 7.22	140.25 ± 7.39	0.83 (0.70–0.99)*	181.13 ± 12.88	175.78 ± 13.04	0.97 (0.80–1.18)	0.24
Total fat (g/day)	91.80 ± 4.41	89.15 ± 4.98	0.97 (0.83–1.12)	82.15 ± 7.19	61.49 ± 5.44	0.73 (0.62–0.86)***	**0.01**
Total water (g/day)	2,988.22 ± 129.68	3,173.98 ± 122.88	1.06 (0.95–1.19)	2,790.44 ± 186.79	2,724.19 ± 190.72	0.98 (0.85–1.13)	0.36
Total dietary fiber (g/day)	27.01 ± 1.75	28.00 ± 2.02	1.04 (0.84–1.23)	24.41 ± 1.96	24.09 ± 1.88	1.00 (0.78–1.27)	0.81
Micronutrients							
Vitamins							
Vitamin A^4^ (mcg RAE/day)	1,974.08 ± 255.78	2,122.00 ± 245.72	1.09 (0.86–1.37)	1,139.47 ± 137.05	1,226.47 ± 207.19	1.10 (0.76–1.61)	0.95
Vitamin C (mg/day)	251.77 ± 30.22	224.47 ± 20.86	1.07 (0.82–1.38)	317.26 ± 62.04	173.93 ± 19.15	0.94 (0.72–1.25)	0.54
Vitamin D^5^ (mcg/day)	67.50 ± 8.89	39.55 ± 6.52	0.54 (0.31–0.93)*	65.76 ± 11.59	53.97 ± 14.87	0.48 (0.12–1.28)	0.86
Vitamin E^6^ (mg/day)	32.42 ± 6.30	23.91 ± 4.47	1.06 (0.85–1.33)	15.01 ± 1.46	10.62 ± 0.99	0.78 (0.65–0.94)**	**0.04**
Vitamin K (mcg/day)	937.93 ± 193.72	1,047.23 ± 183.31	1.12 (0.84–1.49)	523.57 ± 89.99	485.73 ± 71.91	0.93 (0.70–1.25)	0.39
Thiamin (mg/day)	5.25 ± 1.92	6.08 ± 1.84	0.97 (0.80–1.70)	18.79 ± 5.49	18.13 ± 5.26	1.13 (0.85–1.151)	0.37
Riboflavin (mg/day)	3.94 ± 0.94	6.74 ± 1.88	0.99 (0.87–1.12)	19.32 ± 5.47	18.30 ± 5.26	0.90 (0.75–1.08)	0.41
Niacin^7^ (mg/day)	31.26 ± 1.97	32.34 ± 2.61	0.98 (0.86–1.11)	47.95 ± 8.99	45.50 ± 9.08	0.89 (0.67–1.18)	0.57
Vitamin B_6_ (mg/day)	4.52 ± 0.91	8.14 ± 1.88	1.07 (0.90–1.28)	24.39 ± 7.44	23.90 ± 7.66	0.93 (0.72–1.21)	0.38
Folate^8^ (mcg DFE/day)	705.20 ± 57.41	839.80 ± 78.92	1.11 (0.88–1.40)	922.77 ± 149.35	909.44 ± 159.34	1.10 (0.86–1.41)	0.95
Vitamin B_12_ (mcg/day)	312.45 ± 88.48	229.35 ± 50.77	0.93 (0.68–1.27)	214.86 ± 129.64	94.70 ± 46.32	0.62 (0.38–1.04)	0.19
Choline (mg/day)	385.56 ± 17.13	426.53 ± 24.39	1.09 (0.92–1.29)	361.27 ± 42.20	273.20 ± 20.94	0.76 (0.56–1.02)	**0.04**
Minerals							
Calcium (mg/day)	955.71 ± 70.90	858.56 ± 61.09	0.91 (0.74–1.11)	864.31 ± 69.95	766.99 ± 67.48	0.85 (0.72–1.01)	0.62
Copper (mg/day)	2.37 ± 0.28	2.32 ± 0.28	1.02 (0.86–1.22)	1.69 ± 0.13	1.39 ± 0.09	0.85 (0.71–1.01)	0.14
Iron (mg/day)	18.63 ± 1.67	16.30 ± 1.50	0.94 (0.80–1.12)	17.42 ± 2.68	17.11 ± 2.82	0.90 (0.76–1.02)	0.73
Magnesium (mg/day)	493.65 ± 31.83	407.98 ± 24.24	1.00 (0.83–1.21)	423.91 ± 33.73	360.89 ± 24.60	0.92 (0.82–1.04)	0.50
Phosphorus (mg/day)	1,343.77 ± 52.13	1,280.91 ± 63.38	0.95 (0.82–1.10)	1,252.17 ± 88.87	1,071.87 ± 63.42	0.85 (0.70–1.04)	0.39
Selenium (mcg/day)	135.10 ± 13.17	169.69 ± 29.94	1.29 (0.91–1.81)	145.73 ± 20.95	106.83 ± 13.93	0.79 (0.46–1.35)	0.13
Zinc (mg/day)	15.16 ± 1.30	16.05 ± 1.66	0.98 (0.81–1.19)	13.83 ± 2.93	19.74 ± 6.75	0.86 (0.69–1.06)	0.34
Potassium (mg/day)	3,831.98 ± 184.95	3,764.55 ± 177.79	0.98 (0.86–1.13)	3,542.87 ± 262.30	3,366.46 ± 201.71	0.95 (0.80–1.14)	0.78
Sodium (mg/day)	3,302.71 ± 137.22	3,229.49 ± 164.67	0.98 (0.85–1.14)	3,050.60 ± 259.84	2,537.84 ± 219.61	0.83 (0.68–1.01)	0.19

1 Data are shown as mean ± standard error (SE).

2 Proportion of mean change from baseline to 12-months.

3 Significance was determined using generalized linear models with a negative binomial link function.

4 As retinol activity equivalents (RAEs). 1 RAE = 1 μg retinol, 12 μg β-carotene, 24 μg α-carotene, or 24 μg *β*-cryptoxanthin. The RAE for dietary provitamin A carotenoid is two-fold greater than retinol equivalents (REs), whereas the RAE for preformed vitamin A is the same as RE.

5 Vitamin D, D2 + D3 (mcg).

6 Vitamin E, alpha-tocopherol (mg).

7 NE, Niacin Equivalents.

8 DFE, Dietary Folate Equivalents (mcg).

Within-group statistical significance compared to baseline values indicated by * for (*p* ≤ 0.05), ** for (*p* ≤ 0.01), and *** for (*p* ≤ 0.001).

Bold values indicate within-group statistical significance.

**Figure 1:**
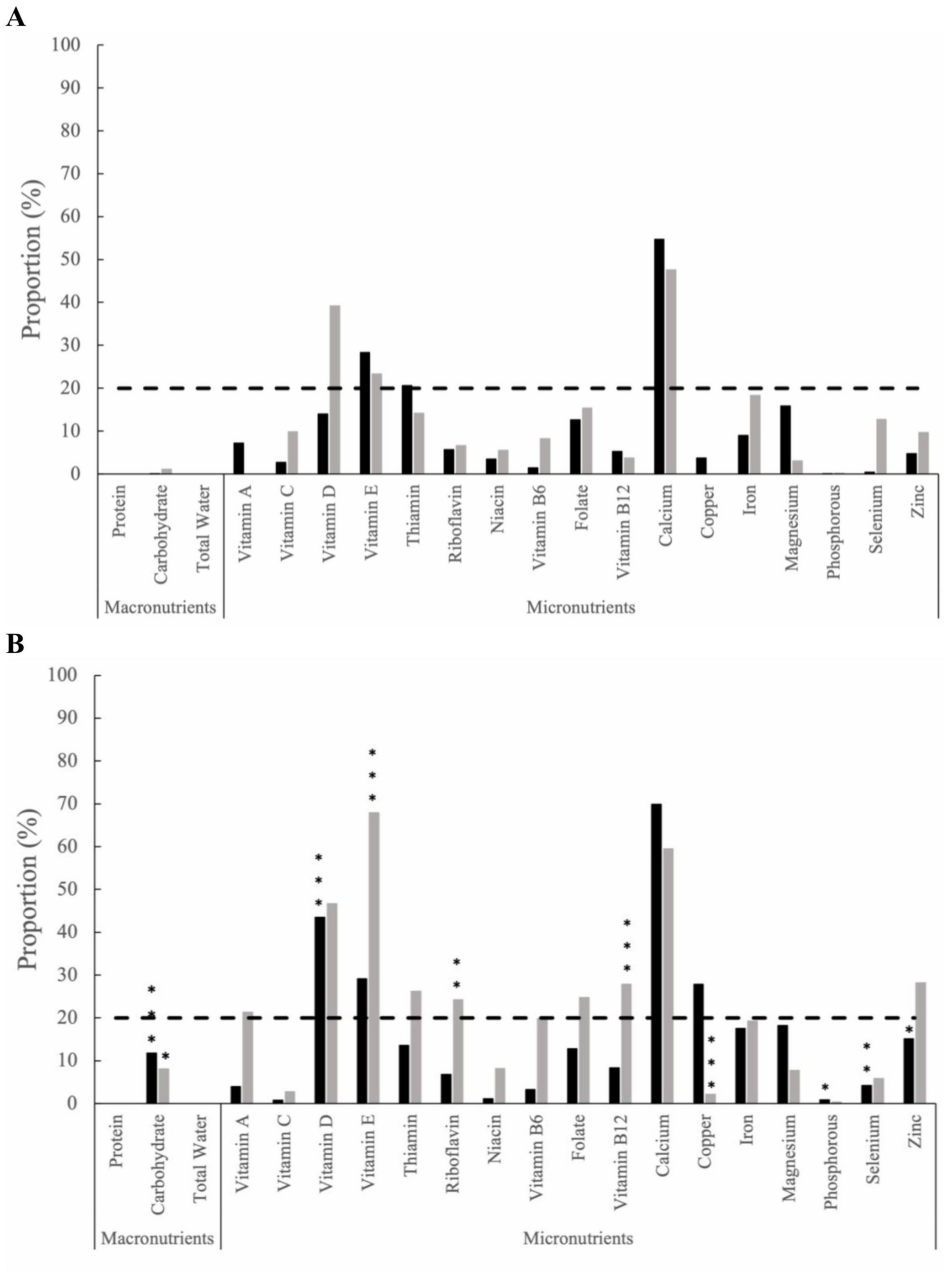
The proportion of inadequate nutrient intake among the health behaviors group (black bars) and standard of care group (grey bars) from food and supplements at **(A)** baseline and **(B)** Month 12. The dashed line represents severe micronutrient inadequate intake, defined as ≥20% of the group. Statistical significance is determined by two sample *z*-tests. Within-group statistical significance compared to baseline values are indicated by * for (*p* ≤ 0.05), ** for (*p* ≤ 0.01), and *** for (*p* ≤ 0.001).

The proportion of inadequate nutrient intake from food and supplements was assessed at baseline (Figure 1A), and within-group comparisons were made throughout the study time intervals. Within the HB group, an increased proportion of inadequate nutrient intake was observed from baseline to month 12 for carbohydrates (11.78%; *p* ≤ 0.001), vitamin D (43.53%, *p* ≤ 0.001), phosphorus (0.82%, *p* = 0.04), selenium (4.17%, *p* ≤ 0.01), and zinc (15.11%, *p* = 0.02) (Figure 1B). In contrast, the SOC group exhibited differing proportions of inadequate nutrient intakes. At 12-months, an increased proportion of inadequate nutrient intake from baseline was observed for carbohydrates (8.10%; *p* = 0.02), vitamin E (67.88%, *p* ≤ 0.001), riboflavin (24.22%, *p* ≤ 0.01), vitamin B12 (27.83%, *p* ≤ 0.001), and copper (2.17%; *p* ≤ 0.001) (Figure 1B). Notably, at 12 months, the HB group exhibited levels above the severe nutrient inadequacy threshold (defined as ≥20% of the group) for calcium (69.88%), vitamin D (43.53%), vitamin E (29.12%), and copper (27.83%). In contrast, in the SOC group, the following micronutrients were found to exceed the severe inadequacy threshold: vitamin E (67.88%), calcium (59.49%), vitamin D (46.64%), zinc (28.18%), vitamin B12 (27.83%), thiamin (26.25%), riboflavin (24.22%), folate (24.75%), and vitamin A (21.27%). An additional proportion of inadequate nutrient intake from food and supplements for months 3, 6, and 9 can be found in the supplemental materials (Supplementary Figure 1).

**Figure 2:**
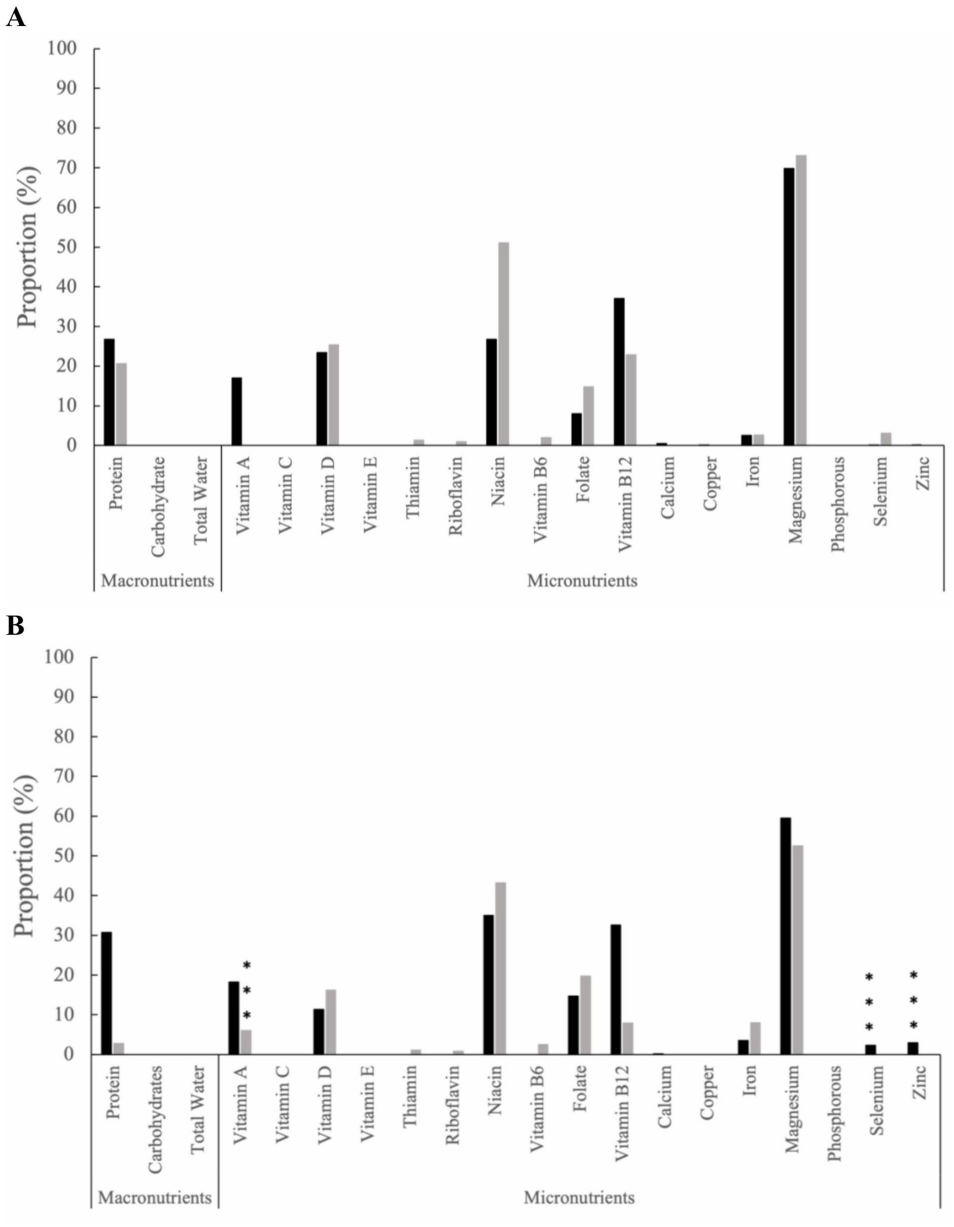
The proportion of excess nutrient intake among the health behaviors group (black bars) and standard of care group (grey bars) from food and supplements at **(A)** baseline and **(B)** Month 12. Statistical significance is determined by two sample z-tests. Within-group statistical significance compared to baseline values are indicated by * for (*p* ≤ 0.05), ** for (*p* ≤ 0.01), and *** for (*p* ≤ 0.001).

Excessive nutrient intake from food and supplements was evaluated from baseline (Figure 2A), with subsequent intra-group comparisons conducted throughout the study time intervals. By 12 months (Figure 2B), the HB group had increased excess nutrient intakes for selenium (2.31%, *p* ≤ 0.001) and zinc (2.92%, *p* ≤ 0.001). In contrast, the SOC group demonstrated excessive nutrient intake solely for vitamin A from baseline to 12 months (6.06%, *p* ≤ 0.001). Additional excessive nutrient intake from food and supplements for months 3, 6, and 9 can be found in the supplemental materials (Supplementary Figure 2).

The feasibility of participants completing the requested 3-day dietary recalls on ASA24 was measured. At baseline, all participants in the HB and SOC groups completed at least 1 day of a 24-h dietary recall, which decreased at the 12-month timepoint, with 95.8% of participants in the HB group and 92.3% of participants in the SOC group completing at least 1 day (Supplementary Table 4). However, the completion percentage of participants declined for the requested 3 days of a 24-h dietary recall using ASA24 for the HB group. At baseline, 86.2% of participants in the HB group and 66.7% of participants in the SOC group completed 3 days of dietary recalls. At 12 months, 83.3% of the participants in the HB group had completed the requested 3 days of 24-h dietary recalls, and 84.6% in the SOC group, surpassing the predefined threshold for success, which was set at greater than 80% completion. There were no statistically significant differences in the percent of completion between the HB and SOC groups at any time point and the number of 24-h recalls completed (*p* > 0.05 for all).

## 4 Discussion

Following a 12-month, remotely delivered, multimodal intervention that included the modified Paleolithic elimination diet, the HB group reduced its intake of added sugars, total grains, refined grains, total dairy, peanuts, tree nuts, and seeds, as well as legumes from baseline to month 12. Moreover, at month 12, using the EAR-cut point method, the proportion of participants with inadequate intake of micronutrients exceeded the threshold for severe inadequacy (defined as ≥20% of the group) in key nutrients, including calcium, vitamin D, vitamin E, and copper. Conversely, within the SOC group, adhering to their typical dietary patterns, the proportion with inadequate intake of micronutrients exceeded the threshold for severe inadequacy for vitamin E, calcium, vitamin D, zinc, and additional nutrients, including vitamin B_12_, thiamin, riboflavin, folate, and vitamin A. These findings suggest that individuals recently diagnosed with RRMS or CIS who adopt a modified Paleolithic elimination diet as part of a lifestyle behavioral intervention may reduce their risk of severe inadequacy for certain nutrients, as compared to adhering to their usual dietary practices.

Participants following the intervention diet significantly reduced their intake of added sugars and total grains while increasing cured meats by 12 months compared to their baseline diet. The observed reductions in food group intakes can be attributed to HB group participants’ high adherence (95.8% adherence) to the modified Paleolithic elimination diet, which has been previously noted (24). The modified Paleolithic diet recommends an intake of meat based on sex and size of the individual, limited intake of gluten-free grains and legumes while strictly avoiding dairy, gluten-containing grains, eggs, and sweeteners/sweet foods, which corresponds with the present study participants’-reduced intake of the respective food groups. Most notably, participants in the HB group consistently maintained a lowered intake of added sugars throughout the entire 12-month duration compared to their baseline usual diet, which was not observed in the SOC group. Added sugars refer to sugars that are added in food preparation or manufacturing, such as glucose, fructose, sucrose (a sugar molecule made from glucose and fructose combined), and hydrogenated starch hydrolysates (high-fructose corn syrup) (43), often used to sweeten or enhance the flavor of foods. Excessive sugar consumption has been associated with various health concerns, including obesity, metabolic disorders, diabetes, cardiovascular disease, cancer, depression, and cognitive impairment (43). Considering the accumulating evidence linking increased added sugar intake and chronic diseases, both the American Heart Association and World Health Organization recommend limiting added sugars to no more than 10% of total calories; which, for the average adult, translates to approximately 200 calories, 50 g, or 12 teaspoons (43), as well as aligning with the modified Paleolithic elimination diet recommendations of one teaspoon or less. Specifically in MS, findings from an animal MS mouse model, experimental autoimmune encephalomyelitis, an animal model of MS, fed a high-fructose diet for up to 12 weeks, found that the abundance of beneficial gut bacteria decreased while potentially pro-inflammatory bacteria were enriched (44). Furthermore, the study revealed increased expression of intestinal immune markers across the small intestine, colon, and spleen (44), suggesting a potential link between high-sugar intake in MS and the alteration of gut microbiota, triggering unfavorable immune responses within both the gut and the periphery, potentially contributing to MS-related symptoms; however, further research is necessary to understand the influence of high-sugar intake among a human MS population. Understanding the food groups eaten by individuals with MS is important, as significant associations of healthy dietary habits, such as increased consumption of fruit, vegetables, and dietary fat food groups, are associated with improved QoL and less likelihood of increased disability when compared to individuals following a less nutritious diet (45).

At 12-months, the HB group exhibited severe inadequacies (>20% of the group) for the micronutrients, vitamin D, vitamin E, calcium, and copper. The micronutrient intakes noted in the HB group share some similarities with a previous randomized control trial involving individuals with RRMS who followed a modified Paleolithic elimination diet, finding that by 12- and 24-weeks, participants had inadequate intake from food and supplements of calcium and iron (22). Since the modified Paleolithic elimination diet restricts dairy, consumption of calcium-fortified foods such as fortified juices or milk alternatives or calcium supplementation may be beneficial (22). Supplementation of vitamin D is recommended for individuals with MS (46), given the lack of vitamin D-rich food. The addition of vitamin E-rich food, such as sunflower seeds, almonds, and avocados, and copper-rich foods, such as oysters and mushrooms, may be beneficial to avoid deficiencies.

Alarmingly, the SOC group, following their own diet, had inadequacies for all the same micronutrients, except for copper, as the HB group, in addition to vitamin A, thiamin, riboflavin, folate, vitamin B_12_, and zinc. This is similar to some of the micronutrients found to be in lower intake among people with MS in the Netherlands, compared to the general population (47). Furthermore, the SOC group inadequacies are further corroborated in the general U.S. adult population, in which inadequacies in vitamin A, vitamin C, vitamin D, vitamin E, calcium, and magnesium intake have been noted both from food and supplements (42). The SOC group’s inadequacies in B vitamins are of concern, as deficiencies in vitamin B_12_ and folate have been linked with increased fatigue (19), lower physical ability (48), and can contribute to the pathogenesis of MS (49). Among individuals with RRMS, vitamin B_12_ and folic acid supplementation have revealed the potential role in improving the QoL (50). In the current study, the supplementation of vitamin B_12_, methyl folate, and pyridoxal-5-phosphate in the HB group may have mitigated deficiencies in these micronutrients, as inadequacies were only present in the SOC group. Thus, suggesting the importance of targeted micronutrient supplementation for individuals with MS to avoid nutrient insufficiencies and that dietary modifications along with nutrient supplementation may benefit individuals with MS (51).

The potential for excess intake of certain nutrients is of concern when combining dietary modifications and nutrient supplementation. By month 12, the HB group had increased excess intake from baseline of selenium and zinc; whereas, the SOC group only had an excess of vitamin A. Excessive intake of certain micronutrients, specifically for fat-soluble vitamins, such as vitamin A, which was observed in the SOC group, are of great concern as accumulation in the body can result in toxicity. On the other hand, excess water-soluble vitamins, such as B vitamins, are of less concern as they can excreted from the body through urine. Mineral excess, such as selenium and zinc observed in the HB group, can result in adverse health events. Selenium toxicity can lead to symptoms such as nausea, vomiting, nail discoloration, hair loss, fatigue, irritability, and foul breath odor (often described as “garlic breath”) (52). Although uncommon, zinc toxicity can lead to nausea, vomiting, epigastric pain, lethargy, and fatigue (53). While no statistically significant changes were observed from baseline to 12 months for both HB and SOC groups, it is noteworthy that magnesium levels exhibited the greatest mineral excess at both time points. This can possibly be attributed to participants taking additional magnesium supplements, given that dietary sources of magnesium, such as dark green vegetable intake, did not change by 12 months and excess. Worth noting, that although excess magnesium intake has not been associated with toxicity, it may lead to gastrointestinal distress and discomfort, such as diarrhea (54). While upper limits of certain nutrients have been created by the Food and Agriculture Organization of the United Nations/World Health Organization to avoid the increased likelihood of adverse effects, excessive intake of nutrients should be monitored, especially in vulnerable populations, such as individuals with MS, to avoid chronic adverse effect outcomes (55).

The feasibility of using ASA24 for conducting 24-h dietary recall among individuals newly diagnosed with MS as a means of dietary assessment was assessed in the present study. Remarkably, at 12 months, both study groups achieved a completion rate of over 80% for the requested 3 days of 24-h recalls; the completion rate increased to above 90% when considering the completion of at least a single 1-day 24-h recall. This high completion rate can potentially be attributed to the proactive approach taken by the Study Coordinator, who sent email reminders to all participants to complete the 24-h recalls at the specific study interval time points, as well as the continuous support from the study RDN for the HB group. While completion of dietary recalls was notably high, it is important to note that three participants required assistance from the study RDN to complete the ASA24 recalls over the phone due to cognitive impairment. While moderate to severe cognitive impairment, as measured by the Short Portable Mental Status Questionnaire (SPMSQ) (26), was an exclusion criterion for the present study, these findings are similar to a prior study that obtained dietary recalls through ASA24 from individuals diagnosed with MS (56). The need for assistance among participants in completing 24-h dietary recalls may be a consideration for future research studies, given that the incidence of cognitive impairment can occur among individuals with MS (57). Potential accommodations could be explored, such as the inclusion of a support individual within the study who could act as a proxy to complete the recalls on behalf of the participant, which could help alleviate the burden associated with recalling dietary intake for individuals with MS. In addition, not all foods and ingredients recommended in the modified Paleolithic elimination diet were available as selectable options within the ASA24 platform. Although suggested food substitutions were provided to HB group participants, this additional step could potentially introduce further participant burden or confusion, particularly for participants with cognitive impairment, as well as not accurately represent the participant’s dietary intake. Searching for alternative objective strategies to assess the dietary intake for people with MS could enhance the accuracy and reliability of dietary assessments for future studies (30).

Strengths of this study include the use of 3-day 24-h dietary recalls and longitudinal collection of dietary recalls; however, limitations were present in this study. The study’s sample size is below the standard recommendation for the NCI method (*n* = 50) used to adjust usual intake; although, the dietary data spanned more days than required by the NCI method to mitigate the impact of limited sample size on variability; thus, these results are not generalizable. Additionally, the ASA24 did not contain several of the foods and ingredients found in modified Paleolithic elimination diet recommendation, which may likely not accurately represent the dietary intake of the HB group participants. Considering the study’s timing during the Coronavirus 2019 (COVID-19) pandemic, it is plausible that participants’ eating habits were influenced (58), and potentially affecting vitamin and mineral supplement intake. Social desirability bias may have resulted in more favorable 24-h dietary intake self-reports from the HB group (59). Furthermore, recall bias may have also influenced the accuracy of usual dietary intake records from all participants (60, 61), especially given that some participants in the current study experienced cognitive impairment. While participants in the HB group were asked about their adherence to each component of the study (i.e., study diet, breathing exercises, and 10-min walking regimen), the vitamin regimen intake adherence was not specifically asked under the study diet, as well as not asked from the SOC group. Future studies should highly consider adding objective measurement methods of dietary intake assessment, in addition to the self-reported ASA24, to ensure accurate analysis.

## In Memoriam

In memoriam of Dr. John Kamholz, who passed during the peer review process of the manuscript.

## Data Availability

The raw data supporting the conclusions of this article will be made available by the authors, without undue reservation.

## Footnotes

^1^ https://asa24.nci.nih.gov
