# Supplementary material for: Feasibility and assessment of self-reported dietary recalls among newly diagnosed multiple sclerosis: a quasi-experimental pilot study
Source: Front Nutr. 2024 Oct 11;11:1369700. doi: 10.3389/fnut.2024.1369700 (PMC11502390; doi:10.3389/fnut.2024.1369700)

Supplementary Material

**Feasibility and assessment of self-reported dietary recalls among newly diagnosed multiple sclerosis: a quasi-experimental pilot study**

**Solange M. Saxby,^1,2^ Mary A. Ehlinger,^1^ Lisa Brooks,^1^ Tyler J. Titcomb,^1^ Patrick Ten Eyck,^3^ Linda M. Rubenstein,^2^ Babita Bisht,^1^ Farnoosh Shemirani,^1^ Christine Gill,^4^ John Kamholtz,^4^ Linda G. Snetselaar,^2,†^ Terry L. Wahls^1^,^†,*^**

*** Correspondence: Terry L. Wahls,** [Terry-Wahls@uiowa.edu](mailto:Terry-Wahls@uiowa.edu)

| **Table S1.** Modified Paleolithic elimination diet food items alternatives for ASA24 suggested to Health Behaviors group participants. | |
| --- | --- |
| **Modified Paleolithic Elimination Diet** | **ASA24 Variable** |
| Any type of leafy green vegetable | Greens (other kind) |
| Any type of liver | Liver |
| Any type of seaweed | seaweed |
| Coconut oil or cream | Oil (unknown kind) |
| Lard, tallow, etc. | Bacon grease |
| Kimchi | Kim chee |
| Any type of seed or nut that cannot be found | Nuts (unknown kind) |
| Herb that cannot be found  (e.g. oregano or basil) | Select a similar herb  (e.g. parsley) |

| **Table S2.** Mean and SE of food groups equivalents at Months 3, 6, and 9 for the Health Behavior and Standard of Care groups.^1^ | | | | | | | |
| --- | --- | --- | --- | --- | --- | --- | --- |
|  | **Health Behavior** | | |  | **Standard of Care** | | |
|  | **Month 3** | **Month 6** | **Month 9** |  | **Month 3** | **Month 6** | **Month 9** |
| ***Fruits Categories (cup eq.)*** |  |  |  |  |  |  |  |
| Total fruits | 1.83 ± 0.14 | 2.13 ± 0.27 | 1.92 ± 0.21 |  | 1.86 ± 0.27 | 2.21 ± 0.21 | 2.30 ± 0.34 |
| Whole or cut citrus, melons, berries (excludes juices) | 0.65 ± 0.09 | 0.72 ± 0.11 | 0.79 ± 0.12 |  | 0.68 ± 0.17 | 0.68 ± 0.16 | 0.49 ± 0.13 |
| Whole or cut other fruits (excludes juices) | 0.95 ± 0.10 | 1.11 ± 0.18 | 0.91 ± 0.13 |  | 0.82 ± 0.17 | 1.13 ± 0.13 | 1.15 ± 0.22 |
| Fruit juices | 0.23 ± 0.04 | 0.30 ± 0.10 | 0.22 ± 0.05 |  | 0.36 ± 0.12 | 0.40 ± 0.12 | 0.66 ± 0.30 |
| ***Vegetables Categories (cup eq.)*** |  |  |  |  |  |  |  |
| Total vegetable (excludes legumes) | 4.78 ± 0.30 | 5.35 ± 0.43 | 5.23 ± 0.37 |  | 3.26 ± 0.31 | 3.58 ± 0.45 | 3.54 ± 0.35 |
| Dark green vegetables | 2.15 ± 0.25 | 2.08 ± 0.32 | 2.11 ± 0.22 |  | 1.12 ± 0.19 | 0.99 ± 0.19 | 1.18 ± 0.16 |
| Total red and orange vegetables) | 0.59 ± 0.08 | 0.73 ± 0.09 | 0.79 ± 0.09 |  | 0.53 ± 0.09 | 0.62 ± 0.11 | 0.67 ± 0.10 |
| Tomatoes and tomato products | 0.13 ± 0.04 | 0.16 ± 0.04 | 0.25 ± 0.06 |  | 0.28 ± 0.07 | 0.34 ± 0.08 | 0.31 ± 0.07 |
| Other red and orange vegetables (excludes tomatoes) | 0.46 ± 0.07 | 0.57 ± 0.09 | 0.54 ± 0.08 |  | 0.25 ± 0.07 | 0.27 ± 0.09 | 0.36 ± 0.10 |
| Total starchy vegetables | 0.22 ± 0.06 | 0.22 ± 0.07 | 0.18 ± 0.05 |  | 0.23 ± 0.08 | 0.46 ± 0.15 | 0.32 ± 0.11 |
| White potatoes | 0.14 ± 0.05 | 0.13 ± 0.06 | 0.10 ± 0.03 |  | 0.19 ± 0.07 | 0.38 ± 0.14 | 0.27 ± 0.10 |
| Other starchy vegetables, excluding white potatoes | 0.08 ± 0.03 | 0.09 ± 0.04 | 0.07 ± 0.03 |  | 0.03 ± 0.02 | 0.08 ± 0.04 | 0.04 ± 0.02 |
| Other vegetables not listed above | 1.82 ± 0.16 | 2.32 ± 0.18 | 2.16 ± 0.22 |  | 1.38 ± 0.17 | 1.52 ± 0.33 | 1.38 ± 0.18 |
| Legumes (beans and peas) computed as vegetables | 0.03 ± 0.01 | 0.03 ± 0.02 | 0.05 ± 0.02 |  | 0.18 ± 0.06 | 0.03 ± 0.01 | 0.08 ± 0.03 |
| ***Grain Category (oz. eq.)*** |  |  |  |  |  |  |  |
| Total grains | 1.19 ± 0.23 | **1.03 ± 0.21 **** | 1.64 ± 0.22 |  | 2.70 ± 0.55 | 3.46 ± 0.52 | 3.55 ± 0.75 |
| Total whole grains | 0.25 ± 0.08 | 0.19 ± 0.08 | 0.16 ± 0.06 |  | 0.38 ± 0.10 | 0.30 ± 0.12 | 0.51 ± 0.23 |
| Total refined grains | **0.94 ± 0.19 *** | **0.83 ± 0.20 *** | 1.48 ± 0.21 |  | 2.33 ± 0.52 | 3.16 ± 0.52 | 3.04 ± 0.71 |
| ***Protein Category (oz. eq.)*** |  |  |  |  |  |  |  |
| Total protein foods (includes eggs, soy, nuts and seeds; excludes legumes) | 10.4 ± 0.65 | 10.8 ± 1.05 | 9.34 ± 0.60 |  | 6.46 ± 0.66 | 5.67 ± 0.49 | 6.29 ± 0.77 |
| Total of meat, poultry, seafood, organ meat, and cured meat | 7.85 ± 0.51 | **8.38 ± 0.98 †** | 7.56 ± 0.59 |  | 4.13 ± 0.59 | **3.70 ± 0.56 †** | 4.89 ± 0.80 |
| Beef, veal, pork, lamb and game meat | 2.33 ± 0.38 | 2.85 ± 0.54 | 1.67 ± 0.38 |  | 1.61 ± 0.45 | 1.93± 0.40 | 1.38 ± 0.41 |
| Cured meats | 0.72 ± 0.17 | 0.69 ± 0.27 | 0.53± 0.16 |  | 0.48 ± 0.22 | 0.36 ± 0.13 | 0.52 ± 0.17 |
| Organ meat | 0.18 ± 0.10 | 0.03 ± 0.02 | 0.21 ± 0.10 |  | 0.05 ± 0.05 | 0.00 ± 0.00 | 0.00 ± 0.00 |
| Poultry | 2.28 ± 0.41 | 2.43 ± 0.48 | 3.38 ± 0.51 |  | 0.95 ± 0.31 | 0.72 ± 0.24 | 1.60 ± 0.42 |
| Eggs and egg substitutes | 0.11 ± 0.06 | **0.05 ± 0.02 *** | 0.33 ± 0.12 |  | 0.18 ± 0.07 | 0.12 ± 0.05 | 0.07 ± 0.03 |
| Soy products excluding soy milk | 0.05 ± 0.05 | 0.00 ± 0.00 | 0.07 ± 0.03 |  | 0.37 ± 0.17 | 0.36 ± 0.16 | 0.15 ± 0.06 |
| Peanuts, tree nuts, and seeds (excludes coconut) | 0.11 ±0.06 | 0.11 ± 0.06 | 0.21 ± 0.09 |  | 0.73 ± 0.26 | 0.11 ± 0.06 | 0.33 ± 0.13 |
| ***Dairy Category (cup eq.)*** |  |  |  |  |  |  |  |
| Total dairy | **0.36 ± 0.06 *** | 0.53 ± 0.10 | 0.39 ± 0.08 |  | 0.82 ± 0.14 | 0.80 ± 0.15 | 0.92 ± 0.18 |
| Milk (includes calcium-fortified soy milk) | 0.25 ± 0.05 | 0.23 ± 0.05 | 0.25 ± 0.06 |  | 0.40 ± 0.09 | 0.29 ± 0.06 | 0.40 ± 0.07 |
| Yogurt | 0.01 ± 0.01 | 0.03 ± 0.02 | 0.03 ± 0.02 |  | 0.07 ± 0.04 | 0.08 ± 0.04 | 0.02 ± 0.02 |
| Cheese | **0.06 ± 0.02 *** | 0.08 ± 0.04 | 0.08 ± 0.04 |  | 0.19 ± 0.06 | 0.29 ± 0.12 | 0.41 ± 0.13 |
| ***Other*** |  |  |  |  |  |  |  |
| Added sugars (tsp. eq.) | **4.74 ± 0.57 **** | **4.76 ± 0.69 *** | **4.54 ± 0.63 *** |  | 7.53 ± 1.45 | 8.29 ± 1.29 | 9.66 ± 1.86 |
| Alcoholic beverages (no. of drinks) | 0.13 ± 0.05 | **0.04 ± 0.04 **** | 0.08 ± 0.04 |  | 0.46 ± 0.26 | 0.52 ± 0.31 | 0.77 ± 0.38 |
| ^1^ Data are shown as mean ± (standard error).  Within-group statistical significance compared to baseline values indicated by ^*^ for (p ≤ 0.05), ^**^ for (p ≤ 0.01), and ^***^ for (p ≤ 0.001).  Between-group magnitude of mean change from baseline statistical significance indicated by † for (p≤ 0.05). | | | | | | | |

| **Table S3.** Mean and SE nutrient intake from food and supplements at Months 3, 6, and 9 for the Health Behavior and Standard of Care groups.^1^ | | | | | | | |
| --- | --- | --- | --- | --- | --- | --- | --- |
|  | **Health Behavior** | | |  | **Standard of Care** | | |
|  | **Month 3** | **Month 6** | **Month 9** |  | **Month 3** | **Month 6** | **Month 9** |
| ***Macronutrients*** |  |  |  |  |  |  |  |
| Energy (kcal/day) | 1,744.31 ± 71.95 | 1,773.69 ± 90.49 | **1,672.35 ± 70.08 *** |  | 1,683.75 ± 95.82 | 1,776.72 ± 92.44 | 1,913.88 ± 128.43 |
| Protein (g/day) | 89.12 ± 4.51 | 98.29 ± 8.16 | 87.19 ± 4.48 |  | 71.62 ± 4.37 | 68.70 ± 4.05 | 73.51 ± 5.42 |
| Carbohydrate (g/day) | 153.33± 7.54 | 165.61 ± 9.72 | 154.93 ± 8.69 |  | 178.50 ± 12.92 | **209.38 ± 10.67 *** | 210.59 ± 18.72 |
| Total Fat (g/day) | 91.69 ± 5.08 | 86.24 ± 6.20 | 83.44 ± 5.23 |  | 76.15 ± 5.97 | 73.98 ± 6.14 | 83.08 ± 6.50 |
| Total Water (g/day) | 3,043.73 ± 139.62 | 3,115.17 ± 155.91 | 3,052.41 ± 166.86 |  | 2,635.12 ± 212.91 | 2,606.20 ± 214.51 | 2,830.54 ± 190.20 |
| Total Dietary Fiber (g/day) | 27.61 ± 1.58 | 30.36 ± 2.35 | 29.35 ± 1.97 |  | 24.35 ± 2.14 | 24.04 ± 2.19 | 23.24 ± 2.15 |
| ***Micronutrients*** |  |  |  |  |  |  |  |
| *Vitamins* |  |  |  |  |  |  |  |
| Vitamin A ^2^ (mcg RAE/day) | 2,334.30 ± 342.97 | 2,079.77 ± 339.70 | 2,310.51 ± 326.20 |  | 1,409.63 ± 210.13 | 979.90 ± 169.30 | 1,140.55 ± 166.13 |
| Vitamin C (mg/day) | 292.69 ± 47.28 | 259.18 ± 25.39 | 259.31 ± 32.92 |  | 168.80 ± 16.95 | 180.84 ± 23.20 | 175.64 ± 24.15 |
| Vitamin D ^3^ (mcg/day) | 66.82 ± 6.44 | 82.28 ± 25.56 | 49.76 ± 6.98 |  | 38.88 ± 8.81 | 35.70 ± 7.42 | 39.23 ± 8.76 |
| Vitamin E ^4^ (mg/day) | 29.16 ±5.67 | 17.70 ± 1.40 | 29.80 ± 6.67 |  | 13.82 ± 1.40 | 12.44 ± 1.21 | 12.14 ± 1.09 |
| Vitamin K (mcg/day) | 1,049.08 ± 227.09 | 1,061.96 ± 299.06 | 982.38 ±155.33 |  | 541.97 ± 94.65 | 389.34 ± 71.56 | 395.61 ± 51.80 |
| Thiamin (mg/day) | 9.17 ± 2.42 | 7.96 ± 2.38 | 5.29 ± 1.89 |  | 13.85 ± 4.91 | 14.38 ± 5.04 | 10.64 ± 4.12 |
| Riboflavin (mg/day) | 10.04 ± 2.44 | 8.71 ± 2.40 | 6.07 ± 1.89 |  | 14.06 ± 4.90 | 14.42 ± 5.04 | 10.82 ± 4.06 |
| Niacin ^5^ (mg/day) | 36.75 ± 2.97 | 34.89 ± 3.37 | 33.77 ± 2.19 |  | 44.40 ± 9.76 | 43.63 ± 9.34 | 35.30 ± 6.93 |
| Vitamin B_6_ (mg/day) | **11.42 ± 2.45 *** | 10.21 ± 2.38 | 7.64 ± 1.88 |  | 21.59 ± 7.86 | 19.87 ± 7.60 | 13.13 ± 5.46 |
| Folate ^6^ (mcg DFE/day) | **986.79 ± 78.02 **** | 863.86 ± 75.85 | 874.23 ± 86.41 |  | 920.61 ± 187.25 | 936.20 ± 172.96 | 712.44 ± 132.68 |
| Vitamin B_12_ (mcg/day) | 347.63 ± 65.43 | 326.63 ± 72.24 | 324.80 ± 75.59 |  | 127.48 ± 48.93 | 91.58 ± 46.45 | 40.57 ± 30.13 |
| Choline (mg/day) | 417.73 ± 21.47 | 441.50 ± 32.45 | **418.74 ± 25.13 †** |  | 300.17 ± 20.03 | 283.96 ± 21.01 | **289.68 ± 21.68 †** |
| *Minerals* |  |  |  |  |  |  |  |
| Calcium (mg/day) | 910.96 ± 78.48 | 918.59 ± 84.53 | 969.24 ± 79.29 |  | 930.66 ± 96.67 | 772.43 ± 80.73 | 811.18 ± 69.33 |
| Copper (mg/day) | 2.94 ± 0.41 | 2.27± 0.23 | 2.71 ± 0.42 |  | 1.80 ± 0.25 | 1.42 ± 0.10 | **1.30 ± 0.10 *** |
| Iron (mg/day) | 15.80 ± 0.98 | 16.17 ± 1.68 | 17.31 ± 1.92 |  | 12.54 ± 0.87 | 12.12 ± 0.73 | 16.03 ± 4.14 |
| Magnesium (mg/day) | 484.35 ± 27.89 | 473.78 ± 38.58 | 469.60 ± 41.23 |  | 364.36 ± 25.41 | 397.40 ± 28.72 | 396.31 ± 34.61 |
| Phosphorus (mg/day) | 1,322.09 ± 58.07 | 1,438.92 ± 96.14 | 1,283.78 ± 53.30 |  | 1,177.49 ± 68.18 | 1,177.11 ± 78.43 | 1,188.76 ± 65.56 |
| Selenium (mcg/day) | 152.92 ± 16.33 | 133.51 ± 16.53 | 170.54 ± 29.50 |  | 134.08 ± 21.45 | 106.36 ± 14.81 | 118.84 ± 17.92 |
| Zinc (mg/day) | 20.17 ± 2.57 | 16.87 ± 1.65 | 18.21 ± 2.06 |  | 11.10 ± 1.20 | 9.33 ± 0.64 | 12.65 ± 3.05 |
| Potassium (mg/day) | 3,883.06 ± 162.35 | 4,289.49 ± 270.41 | 3,872.86 ± 179.48 |  | 3,232.19 ± 205.58 | 3,461.34 ± 265.31 | 3,278.46 ± 239.52 |
| Sodium (mg/day) | **2,923.43 ± 116.77 **** | 3,010.88 ± 214.25 | 3,210.66 ± 199.35 |  | **2,656.33 ± 191.67 *** | 2,714.05 ± 248.97 | 3,001.72 ± 286.97 |
| ^1^ Data are shown as mean ± standard error.  ^2^ As retinol activity equivalents (RAEs). 1 RAE = 1 μg retinol, 12 μg β-carotene, 24 μg α-carotene, or 24 μg β-cryptoxanthin. The RAE for dietary provitamin A carotenoids is two-fold greater than retinol equivalents (REs), whereas the RAE for preformed vitamin A is the same as RE.  ^3^ Vitamin D, D2 + D3 (mcg).  ^4^ Vitamin E, alpha-tocopherol (mg).  ^5^ NE, Niacin equivalents.  ^6^ mcg DFE (Dietary Folate Equivalents).  Within-group statistical significance compared to baseline values indicated by * for (p ≤ 0.05), ** for (p ≤ 0.01), and *** for (p ≤ 0.001).  Between-group magnitude of mean change from baseline statistical significance indicated by † for (p≤ 0.05). | | | | | | | |

| **Table S4.** Feasibility of ASA24 dietary recall completion by participants **^1^** | | | | | |
| --- | --- | --- | --- | --- | --- |
|  | **Baseline** | **3** | **6** | **9** | **12** |
| *Completed the request 3 days of ASA24* | | | | | |
| HB | 86.2 (25) | 60.7 (17) | 48.0 (12) | 45.8 (11) | 83.3 (20) |
| SOC | 66.7 (10) | 60.0 (9) | 66.7 (10) | 69.2 (9) | 84.6 (11) |
| p-value ^2^ | 0.13 | 0.96 | 0.25 | 0.17 | 0.92 |
|  |  |  |  |  |  |
| *Completed at least 2 days of ASA24* | | | | | |
| HB | 96.6 (28) | 82.1 (23) | 60.0 (15) | 62.5 (15) | 87.5 (21) |
| SOC | 93.3 (14) | 66.7 (10) | 73.3 (11) | 69.2 (9) | 92.3 (12) |
| p-value ^2^ | 0.63 | 0.25 | 0.39 | 0.68 | 0.65 |
|  |  |  |  |  |  |
| *Completed at least 1-day of ASA24* | | | | | |
| HB | 100 (29) | 92.9 (26) | 88.0 (22) | 95.8 (23) | 95.8 (23) |
| SOC | 100 (15) | 93.3 (14) | 93.3 (14) | 92.3 (12) | 92.3 (12) |
| p-value ^2^ | 1.00 | 0.95 | 0.30 | 0.65 | 0.65 |
| ^1^ Data are shown as % (n).  ^2^ Significance determined using Pearson’s chi-square test for categorical variables. | | | | | |

**Supplementary Figure Legends**

**Supplementary Figure 1.** The proportion of inadequate nutrient intake among the Health Behaviors group (black bars) and Standard of Care group (grey bars) from food and supplements at **A**) Month 3, **B**) Month 6, and **C**) Month 9. The dashed line represents severe micronutrient inadequate intake, defined as ≥ 20% of the group. Statistical significance is determined by two sample z-tests. Within-group statistical significance compared to baseline values indicated by * for (p ≤ 0.05), ** for (p ≤ 0.01), and *** for (p ≤ 0.001).

**Supplementary Figure 2**. The proportion of excess nutrient intake among the Health Behaviors group (black bars) and Standard of Care group (grey bars) from food and supplements at **A**) Month 3, **B**) Month 6, and **C**) Month 9. Statistical significance is determined by two sample z-tests. Within-group statistical significance compared to baseline values indicated by * for (p ≤ 0.05), ** for (p ≤ 0.01), and *** for (p ≤ 0.001).

**Supplementary Figure 1**

**A**

**
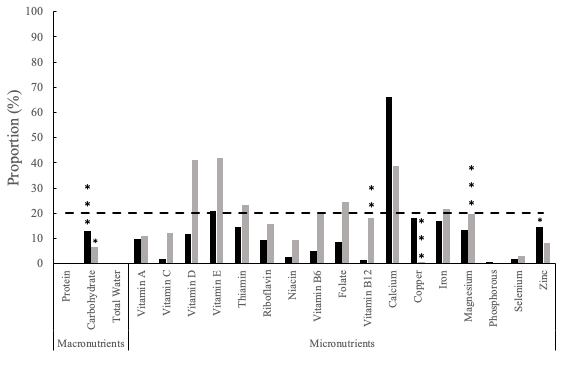
**

**B**

**
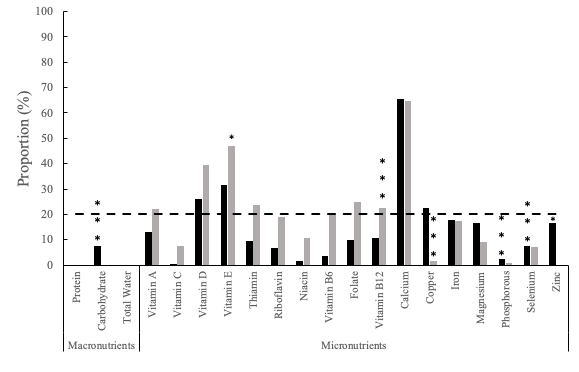
**

**C**

**
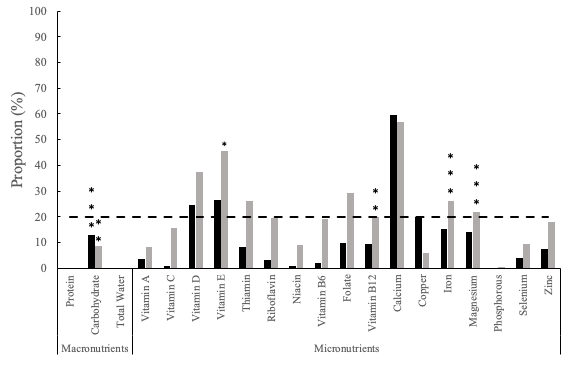
**

**Supplementary Figure 2**

**A**

#
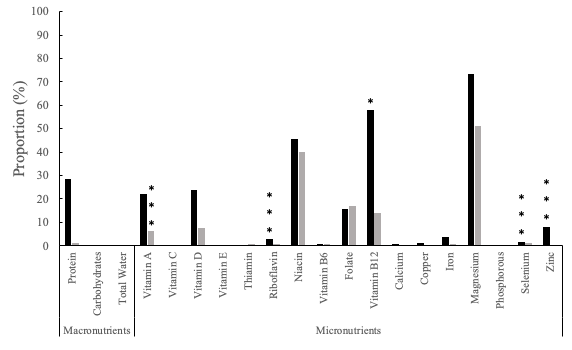


**B**


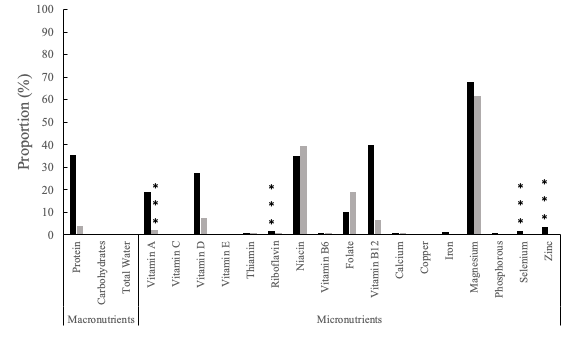


**C**


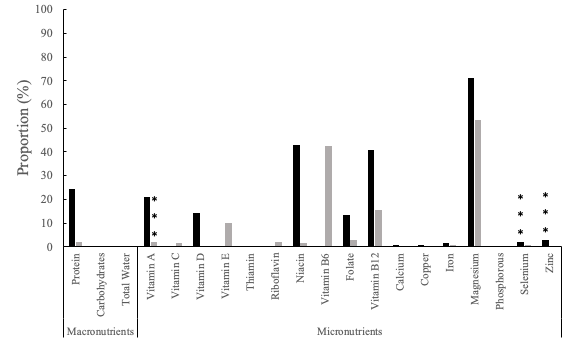

Supplement: Supplementary file 1 [file Table_1.docx]
